# Unveiling a *Salmonella* Enteritidis Outbreak in an Italian Meat Rabbit Farm: Histopathological Features and Epidemiological Investigation

**DOI:** 10.3390/ani15243642

**Published:** 2025-12-17

**Authors:** Giulia Graziosi, Letizia Cirasella, Caterina Lupini, Giulia D’Annunzio, Elena Catelli, Claudio Romboli, Caterina Siclari, Simona Perulli, Laura Fiorentini, Giovanni Tosi, Patrizia Bassi, Giulia Mescolini

**Affiliations:** 1 Department of Veterinary Medical Sciences, University of Bologna, 40064 Ozzano dell’Emilia, BO, Italy; giulia.graziosi2@unibo.it (G.G.); elena.catelli@unibo.it (E.C.); 2 Istituto Zooprofilattico Sperimentale Della Lombardia e Dell’Emilia Romagna (IZSLER), 47122 Forlì, FC, Italy; letizia.cirasella@izsler.it (L.C.); caterina.siclari@izsler.it (C.S.); simona.perulli@izsler.it (S.P.); laura.fiorentini@izsler.it (L.F.); giovanni.tosi@izsler.it (G.T.); 3 Istituto Zooprofilattico Sperimentale Della Lombardia e Dell’Emilia Romagna (IZSLER), 41122 Modena, MO, Italy; giulia.dannunzio@izsler.it; 4 Operating Unit of Animal Health and Hygiene of Livestock Production, Department of Public Health, Azienda Unità Sanitaria Locale (AUSL) della Romagna, 47122 Forlì, FC, Italy; claudio.romboli@auslromagna.it (C.R.); giulia.mescolini@auslromagna.it (G.M.); 5 Istituto Zooprofilattico Sperimentale Della Lombardia e Dell’Emilia Romagna (IZSLER), 40127 Bologna, BO, Italy; patrizia.bassi@izsler.it

**Keywords:** *Salmonella enterica* serovar enteritidis, rabbit meat farming, epidemiological investigation, histopathological features

## Abstract

This study documents an outbreak of *Salmonella enterica* serovar Enteritidis (SE) on a commercial meat rabbit farm in Italy. Following the observation of increased mortality in kits and severe enteric symptoms across all age groups, SE was first isolated in early March 2024. A diagnostic and epidemiological investigation was subsequently undertaken to characterize the anatomo-histopathological features in deceased rabbits and to identify the source and transmission dynamics of the infection. Between March and December 2024, a total of 1550 rectal swabs from live rabbits, 60 environmental samples, and 168 carcasses were collected and subjected to microbiological analysis. SE-positivity rates ranged from 8.4% to 36.3%, depending on the sample type considered. Co-infections with *Pasteurella multocida*, *Escherichia coli*, and *Staphylococcus* spp. were also detected. Gross and histological lesions in SE-positive rabbits included fibrinonecrotizing enterocolitis, hepatosplenomegaly, and renal damage such as suppurative nephritis and tubulorrhexis. Despite the implementation of enhanced biosecurity protocols, SE re-emerged over time and across different pens. Given the zoonotic potential of SE, the outbreak described underscores the need for rabbit-specific *Salmonella* control programs to safeguard both animal and public health.

## Simple Summary

This study reports an outbreak of *Salmonella enterica* serovar Enteritidis (SE) on a commercial meat rabbit farm in Northern Italy, where increased mortality and severe enteric disease prompted investigation. The aim was to characterize the pathological features of clinically diseased rabbits, identify SE infection sources, and understand transmission dynamics. Rectal swabs, environmental samples, and carcasses were analyzed, revealing SE-positivity rates of 8.4–36.3%. Co-infections with *Pasteurella multocida*, *Escherichia coli*, and *Staphylococcus* spp. were also detected. Pathological lesions in SE-positive rabbits included fibrinonecrotizing enterocolitis, hepatosplenomegaly, suppurative nephritis and tubulorrhexis. In the absence of rabbit-specific legislation, control measures were adapted from poultry-based *Salmonella* protocols, yet SE re-emerged despite enhanced biosecurity. Given the zoonotic potential of SE, the outbreak described underscores the need for rabbit-specific Salmonella control programs to safeguard both animal and public health.

## 1. Introduction

Since the primary early civilizations in the Mediterranean basin, rabbits (*Oryctolagus cuniculus*) have been regularly farmed and raised for meat production in Spain, France, Italy, Algeria, and Egypt, as well as in Asian countries like South Korea and China. More commonly farmed and consumed in the past few decades, rabbit meat has showed an estimated overall global reduction in production from approximately 1.2 million metric tons to 681,995 metric tons during the years 2010 to 2023 [1]. Structural weaknesses within the rabbit industry, a progressive and steady drop in consumption, increasing criticism related to animal welfare and other ethical issues have been key factors contributing to this trend [2].

Italian rabbit production, ranking third in Europe following Spain and France, is concentrated in Veneto, Piemonte, Friuli-Venezia Giulia and Lombardia regions, with approximately 4100 farms and 11 million heads per productive cycle [3]. Most of these are small-scale farms, whereas the remaining are closed-cycle commercial farms [4]. From an animal health point of view, intensive farmed rabbits are highly susceptible to gastrointestinal diseases, among which salmonellosis causes serious concern in terms of economic loss and potential risks for public health [5].

Non-typhoidal *Salmonella* spp. (NTSs) has the highest disease burden among human foodborne enteric diseases [6,7]. The Typhimurium and Enteritidis serovars account for the largest number of human infections globally, followed by Infantis, Newport, Dublin, Heidelberg and Weltevreden [7,8,9,10,11]. NTSs are primarily transmitted through consumption of contaminated animal-derived food products or contact with infected animals [12]. Indeed, a broad range of domestic and wild animal species can host *Salmonella* and serve as reservoirs, including poultry, swine, cattle, wild birds, rodents, pets, and exotic animals [13,14,15]. The presence of *Salmonella* in different food matrices varies across countries and regions, depending on cultural and food production practices, geographic location, and economic factors [16]. Pork meat is considered the primary source of human foodborne salmonellosis by the Typhimurium serovar, while *S.* Enteritidis (SE) is found in poultry meat and eggs [11,17]. Current scientific knowledge of *Salmonella* spp. in meat-producing rabbits in Italy is limited, with few reports of *S.* Typhimurium isolation in commercial farms, coupled with the identification of multidrug-resistant strains [18,19,20,21]. This study aims to report an outbreak of SE on a commercial rabbit farm in Northern Italy and to describe the pathological and epidemiological findings documented during the field investigation.

## 2. Materials and Methods

### 2.1. Background

The case study presented began in early March 2024 and was conducted on a meat rabbit farm located in the Emilia-Romagna region, Northern Italy. At that time, the farm housed approximately 6000 does (adult reproductive female rabbits), 17,600 kits (pre-weaning rabbits up to 35 days of age) and 20,200 fattening rabbits (rabbits up to 75–85 days of age), reared in eight pens: four fattening pens (pens 1 and 2 with bicellular cages, pens 3 and 4 with enriched cages designed according to the World Rabbit Science Association guidelines), and four breeding/fattening pens (pens 5 to 8, equipped with dual-purpose cages).

Clinical signs were reported in weanling kits and does in pen 5, with the former exhibiting depression and high mortality rates, and the latter showing severe enteric symptoms. Following the post-mortem examination of 16 carcasses submitted to the Istituto Zooprofilattico Sperimentale della Lombardia e dell’Emilia-Romagna (Forlì, Emilia-Romagna, Italy) for diagnostic purposes, SE was detected. A thorough diagnostic and epidemiological investigation was therefore conducted to characterize the anatomo-histopathological features of diseased rabbits, and to elucidate the source and exposure of the infection.

### 2.2. Microbiological Investigation

Samples to be submitted for microbiological analyses were collected over the course of 19 farm visits; all the analyses were conducted at the Istituto Zooprofilattico Sperimentale della Lombardia ed Emilia-Romagna (Forlì, Italy).

Rectal swabs were collected from individual live rabbits and pooled in groups of 10. Pooled samples were processed according to the ISO 6579:2002/Amd 1:2007 method for *Salmonella* spp. [22]. Briefly, swabs were transferred to 10 mL of Buffered Peptone Water (Biolife Italiana s.r.l.—Mascia Brunelli S.p.A, Milano, Italy) and incubated at 37 °C for 24 h. Thereafter, 0.1 mL of the pre-enrichment was inoculated onto a Modified Semisolid Rappaport Vassiliadis (Oxoid, Hampshire, UK) medium and incubated at 41.5 °C for 48 h. Suspect colonies were plated onto Xylose Lysine Deoxycholate agar (bioMérieux, Bagno a Ripoli, Italy) and brilliant green agar (Vacutest Kima, Arzergrande, Italy) and incubated at 37 °C for 24 h. Microbiological analyses were also performed on any carcasses collected during farm visits. Multiple organs (brain, intestine, lungs, liver, kidneys, and spleen) were collected for standard microbiological examination [23]. Briefly, organ samples were inoculated on blood agar plates, whereas Hektoen enteric agar was used for the selective growth of Enterobacterales and Pseudomonadales. Both types of plates, then, were incubated at 37 °C for 48 h under both aerobic and anaerobic conditions.

Contents of the large intestine were collected and analyzed as previously mentioned for rectal swabs [22].

To assess the efficacy of cleaning and disinfection procedures of empty pens or to assess the degree of SE environmental contamination during outbreaks, 5 environmental samples per pen were collected (cage surfaces, waterers, feeders) through sterile sponge-sticks and individually analyzed according to the above-mentioned ISO 6579:2002/Amd 1:2007 method for *Salmonella* spp. [22].

All presumptive *Salmonella* spp. isolates from rectal swabs, environmental samples, and carcasses were confirmed using suitable biochemical tests (EnteroPluri-Test, Liofilchem^®^, Roseto degli Abruzzi (TE), Italy). Serological confirmation of the *Salmonella* strains was performed in accordance with the ISO 6579-3:2014 [24], following the Kauffmann–White scheme.

### 2.3. Post-Mortem Examination and Histopathology

During the interventions, post-mortem examination was conducted on carcasses in good conservation status. Of these, a subset of diseased rabbits of different ages was randomly selected, and their organs were submitted for histopathology. Specifically, samples of liver, spleen, kidneys, lungs, heart, intestine, and brain were collected, fixed in 10% buffered formalin, and processed according to standard histological procedures [25]. Four-micrometer sections were obtained from formalin-fixed paraffin-embedded (FFPE) tissue blocks and stained with hematoxylin and eosin (H&E) for microscopic evaluation of pathological changes.

### 2.4. Epidemiological Investigation and Control Measures

An epidemiological investigation was conducted to clarify the potential source and exposure of SE infection. The proper implementation of biosecurity measures was assessed through a questionnaire administered to the farmer by the Veterinary Competent Authority. This included questions on farm’s structural features and management practices. Given the lack of rabbit-specific legislation, epidemiological investigation and control measures of the outbreaks were adapted from the Italian salmonellosis control guidelines in poultry [26].

## 3. Results

### 3.1. Microbiological Investigation

Over the course of the investigation, SE was detected in rabbits of all ages housed in different pens. The results of the microbiological analyses are summarized in Table 1.

**Table 1:** Results of microbiological investigation in meat rabbit farm.

Sample Type	Date	Number of Samples Analyzed	Number of SE-Positive Samples (%)	Pen Sampled (SE-Positive Pen; Organ)	Other Bacteria Isolated (Pen)
Carcasses (first detection)	5 March 2024	16	16 (100)	5 (5; liver, brain, spleen)	*Pasteurella multocida* in lungs (5)
Carcasses	18 March 2024	20	5 (25)	5, 6, 7, 8 (5; liver, brain, spleen)	*Pasteurella multocida* in lungs (7)
Rectal swabs (pooled)		20	1 (5)	5, 6, 7, 8 (7)	n.a. *
Carcasses	19 March 2024	20	0 (0)	1, 2, 3, 4 (n.a.)	Systemic *E. coli* (2); *P. multocida* in lungs and liver (4)
Rectal swabs (pooled)		20	0 (0)	1, 2, 3, 4 (n.a.)	n.a.
Carcasses	9 April 2024	5	0 (0)	7 (n.a.)	n.a.
Rectal swabs (pooled)		5	0 (0)	7 (n.a.)	n.a.
Environmental samples (C&D) ^A^	26 April 2024	5	0 (0)	5 (n.a.)	n.a.
Carcasses	10 May 2024	5	0 (0)	5 (n.a.)	n.a.
Rectal swabs (pooled)		5	3 (60)	5 (5; n.a.)	n.a.
Rectal swabs (pooled)	29 May 2024	10	0 (0)	5, 7 (n.a.)	n.a.
Environmental samples (C&D)	13 June 2024	5	0 (0)	7 (n.a.)	n.a.
Carcasses	22 August 2024	17	7 (41.2)	5, 7 (5; spleen and liver)	Systemic *P. multocida* (7)
Carcasses	3 September 2024	38	18 (47.4)	1, 2, 3, 4, 5, 6, 7, 8 (1; liver, brain, spleen; 5; intestine; 6; intestine; 7; liver, brain, spleen)	*Staphylococcus* spp. in kidneys, liver and lungs, (1); systemic *E. coli* (2, 3, 5); *P. multocida* in lungs (2).
Rectal swabs (pooled)		40	7 (17.5)	1, 2, 3, 4, 5, 6, 7, 8 (1, 5, 6)	n.a.
Environmental samples (Con) ^B^	11 September 2024	25	18 (72)	1, 2, 3, 4, 5, 6, 7, 8 (1, 2, 5, 6, 7)	n.a.
Carcasses	27 September 2024	5	0 (0)	5 (n.a.)	n.a.
Rectal swabs (pooled)		5	0 (0)	5 (n.a.)	n.a.
Carcasses	18 October 2024	5	0 (0)	7 (n.a.)	n.a.
Rectal swabs (pooled)		10	0 (0)	7 (n.a.)	n.a.
Carcasses	6 November 2024	5	5 (100)	6 (6, intestine)	n.a.
Rectal swabs (pooled)		5	0 (0)	6 (6)	n.a.
Carcasses	21 November 2024	10	10 (100)	1, 2 (1, intestine; 2, intestine)	*P. multocida* in lungs (1, 2)
Rectal swabs (pooled)		10	2 (20)	1, 2 (2)	n.a.
Carcasses	25 November 2024	4	0 (0)	6 (n.a.)	n.a.
Rectal swabs (pooled)		5	0 (0)	6 (n.a.)	n.a.
Carcasses	29 November 2024	5	0 (0)	8 (n.a.)	*P. multocida* in lungs (8)
Rectal swabs (pooled)		5	0 (0)	8 (n.a.)	n.a.
Environmental samples (C&D)	4 December 2024	25	0 (0)	3, 5, 6, 7, 8 (0)	n.a.
Carcasses	11 December 2024	8	0 (0)	1, 4 (n.a.)	n.a.
Rectal swabs (pooled)		10	0 (0)	1, 4 (n.a.)	n.a.
Carcasses	19 December 2024	5	0 (0)	2 (n.a.)	*P. multocida* in lungs (2)
Rectal swabs (pooled)		5	0 (0)	2 (n.a.)	n.a.

* n.a. not applicable. ^A^ Samples collected following cleaning and disinfection procedures of empty pens (C&D). ^B^ Samples collected to assess the degree of environmental contamination during outbreaks (Con).

A total of 1550 rectal swabs were obtained; 8.4% (13/155) pooled swabs tested positive for SE isolation. 168 carcasses were also collected and analyzed microbiologically, with an overall positivity rate of SE of 36.3%. Other bacteria isolated in different organs included *Pasteurella multocida*, *Escherichia coli*, and *Staphylococcus* spp., either as single agents or in mixed infections, sometimes in association with SE. With respect to environmental samples, a total of 60 were collected and 30% tested positive for SE isolation. All positive samples were among those collected to assess SE environmental contamination (Con) during outbreaks, whereas samples collected after cleaning and disinfection (C&D) tested negative.

### 3.2. Post-Mortem Examination

#### 3.2.1. Gross Pathology

Necropsies were performed on 119 carcasses; the remaining specimens were unsuitable for post-mortem examination due to poor preservation and advanced carcass degradation.

**Figure 1:**
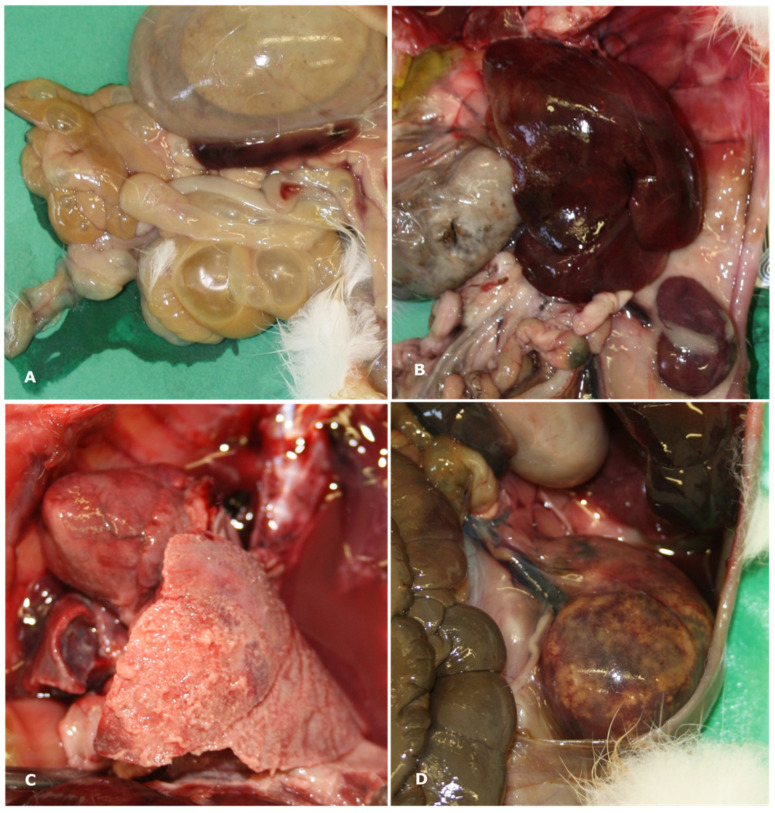
Macroscopic findings observed during necropsy included (**A**) dilation of the gastrointestinal tract with severe catarrhal enterocolitis; (**B**) severe hepatosplenomegaly and congestion; (**C**) severe, diffuse fibrinosuppurative pleuropneumonia; and (**D**) suppurative glomerulonephritis.

The main gross findings observed are presented in Table 2. In individuals that tested positive to SE, gross lesions were primarily observed in the intestine, liver, and spleen. Intestinal dilation and catarrhal enterocolitis were observed in 57.5% of cases (Figure 1A), whereas 15.5% exhibited hemorrhagic enterocolitis. Hepatomegaly (39.1%) and splenomegaly (27.9%) (Figure 1B), fibrinous perisplenitis (10.5%), and hepatic congestion (3.1%) were also detected. Pulmonary involvement was observed in 65.2% of the rabbits examined, including: pulmonary edema (12.4%), pulmonary hemorrhages (32.2%), suppurative pneumonia (9.3%), and fibrinosuppurative pleuropneumonia (11.1%) (Figure 1C). Tracheal hyperemia was present in 13.6% of cases. Cardiomegaly, pericardial effusion, and epicardial hemorrhages were observed in a small percentage of cases (1.2%, 0.6%, and 6.2%, respectively). Interstitial nephritis and suppurative glomerulonephritis were each identified in 0.6% of cases (Figure 1D).

**Table 2:** Key gross lesions observed at necropsy in Salmonella Enteritidis (SE)-positive and -negative cases. Additionally, other bacteria isolated from carcasses are listed.

Gross Lesions Observed	Number of SE-Positive Individuals	Number of SE-Negative Individuals	Other Bacteria Isolated
Catarrhal enterocolitis	35	57	*P. multocida*, *E. coli*
Hemorrhagic enteritis	15	10	*E. coli*
Splenomegaly	10	35	*P. multocida*, *E. coli*
Fibrinous perisplenitis	7	10	*P. multocida*
Hepatomegaly	23	40	*P. multocida*, *E. coli*
Hepatic congestion	5	0	*E. coli*
Pulmonary edema	0	20	*P. multocida*, *E. coli*
Pulmonary hemorrhages	12	40	*P. multocida*, *E. coli*
Suppurative penumonia	5	10	*P. multocida*
Fibrinosuppurative pleuropneumonia	5	13	*P. multocida*
Tracheal hyperemia	7	15	*P. multocida*
Cardiomegaly	0	2	n.a. *
Pericardial effusion	0	1	n.a.
Epicardial hemorrhages	0	10	*E. coli*
Interstitial nephritis	0	1	*Staphylococcus* spp.
Suppurative glomerulonephritis	1	0	n.a.

* n.a., not applicable.

#### 3.2.2. Histopathology

Tissue samples for histological analysis were obtained from five animals from pen number 1 (Animal ID: 271429, kit), 2 (ID: 271461, fattening rabbit at the start of cycle), 4 (ID: 271463, fattening rabbit), 5 (ID: 271474, fattening rabbit), and 7 (ID: 271479, doe). Three of five animals from which tissues were collected for histological examination tested positive for SE (Animal IDs: 271474, 271429, 271479), either alone or in co-infection with hemolytic *Staphylococcus* spp. (ID: 271429) or *E. coli* (ID: 271474).

**Figure 2:**
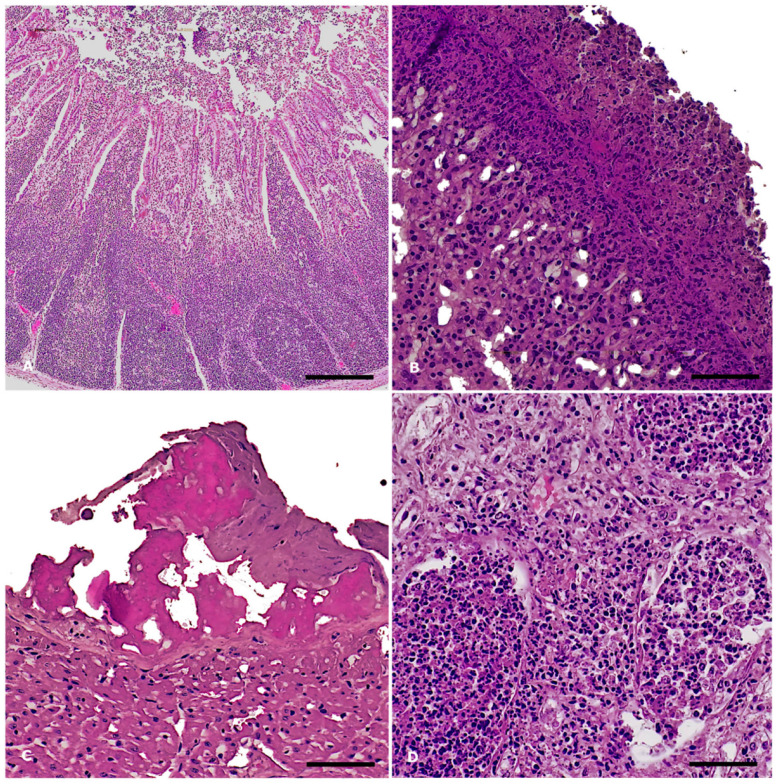
*Salmonella* Enteritidis outbreak in rabbits: histopathological findings, *S*. Enteritidis-positive cases. Hematoxylin and eosin stain. (**A**) Ileum: severe, diffuse, acute fibrinonecrotizing enterocolitis and Peyer’s patches hyperplasia. Magnification: 4X Scale bar: 800 µm; (**B**) Lung: severe, acute fibrinosuppurative pleuropneumonia. Magnification: 20X. Scale bar: 100 µm; (**C**) Heart: severe, acute fibrinous pericarditis. Magnification: 20X. Scale bar: 200 µm; (**D**) Kidney: severe, acute suppurative nephritis with tubulorrhexis. Magnification: 20X. Scale bar: 200 µm.

A consistent finding across all three cases was severe fibrinonecrotizing enterocolitis. The lamina propria was heavily infiltrated by lymphocytes and plasma cells, interspersed with macrophages and karyorrhectic heterophils (Figure 2A). The mucosal surface exhibited multifocal erosion and ulceration, characterized by enterocyte necrosis and desquamation of epithelial cells into the lumen, and was effaced by cellular debris admixed with fibrin. In all cases, the spleen exhibited marked erythrocytic engorgement (congestion). Hepatic congestion was noted in all three animals, with mild bile ducts hyperplasia observed in two cases (IDs: 271474, 271479), while one animal (ID: 271429) presented with centrilobular hepatic degeneration, likely of hypoxic origin. Pulmonary congestion and edema were evident in all three cases. Notably, in animal ID 271429 the pleurae were thickened by extensive fibrin deposition associated with numerous karyorrhectic heterophils and scattered bacterial aggregates, with a similar inflammatory infiltrate admixed with fibrin expanding the alveolar spaces of the subpleural pulmonary parenchyma, consistent with fibrinosuppurative pleuropneumonia (Figure 2B). In this animal, fibrinosuppurative inflammation was also observed in the pericardium (Figure 2C). Renal pathology in all three animals included acute tubular degeneration and necrosis. Additionally, the kidney of animal ID 271479 exhibited multifocal tubulorrhexis with aggregates of karyorrhectic heterophils in the renal tubular lumens and in the interstitium (Figure 2D). Cerebral vascular hyperemia was a consistent finding in all three animals.

**Figure 3:**
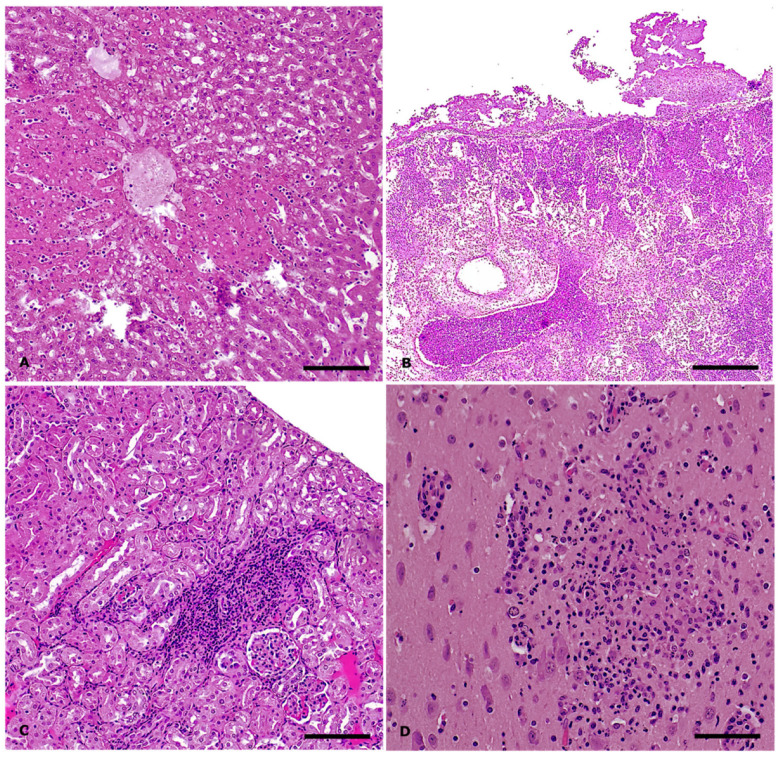
*Salmonella* Enteritidis outbreak in rabbits: histopathological findings, *S*. Enteritidis-negative cases. Hematoxylin and eosin stain. (**A**) Liver: severe, acute, centrilobular hepatic necrosis in systemic *Pasteurella multocida* infection. Magnification: 10X Scale bar: 200 µm; (**B**) Lung: severe, acute, suppurative bronchopneumonia and fibrinous pleuropneumonia in systemic *P. multocida* infection. Magnification: 4X. Scale bar: 500 µm; (**C**) Kidney: moderate, chronic lymphoplasmacytic tubulointerstitial nephritis. Magnification: 10X. Scale bar: 200 µm; (**D**) Brain: focal neuropil effacement by inflammatory cells and cellular debris in case of lymphoplasmacytic and histiocytic meningoencephalitis. Magnification: 20X. Scale bar: 50 µm.

The remaining two subjects from which tissue samples were collected for histological examination were negative for *Salmonella* spp. However, one (Animal ID: 271461) tested positive for *P. multocida* and *E. coli*, while the other (Animal ID: 271463) tested negative at the microbiological analysis). Histologically, the case with systemic *P. multocida* infection presented with hepatic necrosis exhibiting a centrilobular distribution (Figure 3A), splenic congestion, and chronic lymphoplasmacytic enteritis with hyperplasia of Peyer’s patches. Pulmonary and cardiac examination revealed thickening of the serosal membranes by abundant fibrin intermingled with aggregates of karyorrhectic heterophils. In the lungs, the lumens of the airways were filled with abundant exudate composed of karyorrhectic neutrophils (Figure 3B). Mild gliosis and hyperemia were observed in the brain. The remaining animal (Animal ID: 271463) presented with necrotizing enteritis and mild cholangitis in the liver, accompanied by slight bile duct hyperplasia and lymphoid depletion in the spleen. The most significant microscopic lesions were observed in the kidney and brain. Approximately 40% of the renal parenchyma exhibited chronic lymphoplasmacytic interstitial inflammation associated with acute tubular necrosis and degeneration, consistent with tubulointerstitial nephritis (Figure 3C). In the brain, the findings were consistent with lymphoplasmacytic and histiocytic meningoencephalitis (Figure 3D). Throughout the brain parenchyma, Virchow-Robin spaces were expanded by a moderate number of macrophages, lymphocytes, and plasma cells, along with scattered heterophils (perivascular cuffing). Additionally, the neuroparenchyma displayed scattered multifocal foci characterized by aggregates of macrophages and heterophils, as well as multifocal aggregates of extravasated erythrocytes (hemorrhage).

### 3.3. Epidemiological Investigation and Control Measures

With respect to farm location, it was situated in a rural area of the Forlì province, Emilia-Romagna region. The owner of the investigated farm possessed other rabbit holdings located within a radius of 40 km. None of these reported any *Salmonella*-related clinical signs during the outbreak. Two separate commercial broiler farms were located within a 1 km radius; according to surveys conducted under the Italian salmonellosis control guidelines in poultry, neither reported any SE cases [26].

Regarding the farm’s structural features, the floors and walls of the pens were cleanable and washable. A forced longitudinal ventilation system was installed. The surrounding area, within two meters of the pens, was paved, washable, and regularly maintained in a clean condition.

A core staff of four permanent employees operated in the farm and were responsible for animal care. During weekends, only one staff member was present. Farm workers also performed artificial insemination of does. Water was provided ad libitum for all rabbits and sourced from aqueduct. Biosecurity measures in place are summarized in Table 3.

**Table 3:** List of biosecurity measures in place in the farm.

Biosecurity Measure	Actions in Place
Feed and water	A total of 16 silos were used for feed storage; they remained sealed and showed no signs of breaches. The water was sourced from the aqueduct and was monitored annually for microbiological quality.
Management of manure and carcasses	Rabbit manure was regularly removed from pens and temporarily stored on the farm premises before being transported to an external biogas production facility. Animal carcasses were collected daily and stored in a dedicated freezer located in a separate room within farm premises. A licensed rendering company accessed the farm to retrieve the carcasses for proper disposal.
Management of equipment	Equipment, including tools for cleaning and disinfection, and artificial insemination, was used across multiple pens, without any record of its movements.
Visitors and farmworkers	Access by unauthorized individuals was restricted through a physical barrier installed at the farm entrance. A visitor logbook was maintained, and personal protective equipment (PPE) was provided to all external visitors. Farm personnel were partially shared with other rabbit production units under the same ownership. Upon entry, workers were required to change into farm-specific clothing. No hygiene locks or disinfection stations were present at the farm entrance or at pen entry points, and footbaths were not implemented at pen access areas.
Pest control	A rodent control program was implemented and documented through a written operating procedure. Bait stations were routinely monitored to assess consumption levels. However, systematic review of program outcomes and a formal procedure for periodic updates were not established.
Cleaning and disinfection	Written operating procedures for pen cleaning and disinfection were not established. However, cages were cleaned and disinfected after being vacated and prior to the introduction of a new rabbit batch. An automated vehicle disinfection system, consisting of a disinfection arch for trucks, was installed and operational at the farm entrance.
Presence of other animals	A dog owned by the farm manager was present within the farm premises. Feral cats were observed on site, and pigeons were frequently sighted by farm personnel.

Given the detection of *P. multocida* and the presence of respiratory symptoms, affected does, juveniles, and fattening rabbits were treated via drinking water using a rotational protocol prescribed by the farm veterinarian. Sulfadimethoxine–trimethoprim (1 mL/10 kg), oxytetracycline (40 mg/kg), or enrofloxacin (10 mg/kg) were used. Sampling collection was performed before any administered treatment.

In the absence of specific regulations for rabbits, control measures for SE were adapted from Italian salmonellosis control guidelines in poultry [26]. SE-positive rabbits were placed under official detention by the Veterinary Competent Authority, in accordance with Regulation (EU) 2017/625. Following the resolution of SE-related clinical signs and, where applicable, the completion of the above-mentioned antibiotic withdrawal period, animals were deemed eligible for slaughter only after testing negative for *Salmonella* spp. on rectal swabs collected by an Official Veterinarian. After the transport of fattening rabbits to the slaughterhouse, cages were cleaned and disinfected using a combination of glutaraldehyde- and chlorine-based formulations. To assess the effectiveness of C&D procedures, environmental samples were collected post-cleaning and disinfection, as previously described (results in Table 1).

Throughout the investigation period, enhanced biosecurity measures were implemented. These included the use of disposable Personal Protective Equipment (PPE) by all personnel entering the facility; biweekly manure removal from occupied pens and cleaning and disinfection of vehicles used for manure transport; thorough cleaning and disinfection of equipment, materials and storage rooms; accurate manure removal from empty pens, followed by cleaning and disinfection; and monitoring the effectiveness of the rodent control plan. Deceased animals were placed in sealed plastic bags, stored in a closed washable container outside the pen, and collected by a designated individual.

## 4. Discussion

This study documents an outbreak of SE, a zoonotic pathogen of significant public health relevance, in a commercial rabbit farm in Northern Italy. *Salmonella* spp. is recognized as a primary pathogen in rabbits, frequently associated with high morbidity and mortality rates in the affected groups [21,27,28,29]. Among the different serovars, *S.* Typhimurium has been especially reported in clinically diseased rabbits [19,21,30,31,32,33,34]. Although SE has been less frequently reported [34,35], this study highlights its potential to cause disease. The infection presented as an acute clinical condition characterized by elevated mortality in kits and severe enteric symptoms in rabbits of all ages, with SE extensively detected across multiple pens over a prolonged period. The concurrent isolation of *P. multocida* and other bacterial pathogens indicates a multifactorial pathological scenario, where co-infections likely exacerbated disease severity.

From a pathological perspective, the gross and histological findings associated with SE infection included severe fibrinonecrotizing enterocolitis, hepatosplenomegaly, and pleuropneumonia. These were consistent with the systemic nature of salmonellosis in rabbits [36,37]. Considering the pathological effects of *Salmonella* spp. on renal tissue [38], the observed kidney lesions, particularly suppurative nephritis and tubulorrhexis, may represent underreported consequences of severe septicemia in lagomorphs.

In the reported outbreak, the precise source of infection remained unidentified, which limited the effectiveness of the implemented outbreak control measures. The primary introduction of SE through wild animal reservoirs (e.g., rodents, wild birds) cannot be excluded, nor can the presence of a persistent environmental or animal reservoir that may have contributed to the repeated detections over time. Although contaminated feed or water are potential sources of *Salmonella* spp. in farmed animals [39,40,41], these were not specifically sampled during the outbreak, representing a limitation in fully assessing all possible routes of SE entry. Given the persistence of *Salmonella* spp. in the environment [21,42], the implementation of strict biosecurity protocols, along with thorough cleaning and disinfection of pens and cages, is essential to minimize the risk of contamination and intra-farm transmission [43,44]. The widespread detection of SE in both live and deceased rabbits across multiple pens suggests that biosecurity measures within the farm were either inadequately applied or insufficiently enforced. Although certain control practices were in place, several critical biosecurity gaps were identified. These included the lack of equipment tracking and its shared use across pens, as well as the presence of shared personnel responsible for animal care and reproductive procedures, which may have facilitated cross-pen SE transmission. Notably, rabbit and broiler farms located nearby remained negative for SE, suggesting that the outbreak was successfully confined to the affected premises.

Despite the extensive diagnostic effort inherent to this field study, some limitations should be acknowledged. While pooling can increase the efficiency of microbiological testing [45], it can reduce the sensitivity of detection due to a dilution effect [46,47]. Therefore, the positivity reported for pooled swabs may have underestimated the true SE status of individual rabbits. Additionally, molecular characterization of SE isolates was beyond the scope of this study and would have provided further insights into strain relatedness and transmission dynamics [48,49].

In Italy, *S.* Enteritidis is considered the one of the most frequently isolated *Salmonella enterica* subsp. *enterica* serovar in food-producing animals, alongside with the Infantis and Typhimurium serovars [50]. The absence of surveillance for *Salmonella* spp. in rabbit meat farming represents a significant gap in current zoonotic disease control strategies. Although rabbit meat is typically consumed cooked, reducing the risk of foodborne infection, improper handling or cross-contamination during processing may still pose a zoonotic hazard [51]. Future investigations should therefore prioritize longitudinal surveillance of *Salmonella* spp. in farmed rabbits, along with the assessment of species-specific biosecurity and control measures, to improve the prevention and management of *Salmonella* infections in commercial rabbit production systems.

## 5. Conclusions

This study demonstrates the pathogenic potential of SE in commercial meat rabbits and highlights the critical influence of farm management and biosecurity in shaping outbreak dynamics. Given the zoonotic risk associated with SE and its persistence in intensive farming systems, surveillance programs that include *Salmonella* spp. in rabbit production should be prioritized by regulatory authorities. Furthermore, the development of rabbit-specific control measures, tailored to the unique characteristics of this production system, would be essential to safeguard both animal and public health.

## Data Availability

All data are included within the manuscript.
